# Honoring Older Latino Immigrants’ Perspectives on Cancer: Informing the Future

**DOI:** 10.1093/geroni/igaa057.2404

**Published:** 2020-12-16

**Authors:** Iraida Carrion, Malinee Neelamegam, Jane Roberts

**Affiliations:** 1 University of South Florida, Tampa, Florida, United States; 2 Yale School of Public Health, New Haven, Connecticut, United States; 3 University of South Florida, Sarasota, Florida, United States

## Abstract

Cancer is the leading cause of death among Latinos in the U.S. Approximately 32.2% of Latinas and 44.1% of Latinos aged 60 years or older have a lifetime probability of developing invasive cancer (ACS, 2018), with lower survival rates for most cancers even when allowing for age and stage distribution. There is some evidence that older Latino/Latina immigrants lack knowledge about cancer treatment options and are often adversely impacted by healthcare inequities regarding cancer treatment and care options. This study compared the cancer beliefs and attitudes of 58 Latinos and 110 Latinas with a mean age of 67.9 years who reside in the Greater Tampa Bay area. Recruitment occurred in community-based settings, and interviews were conducted in Spanish and transcribed into English. The qualitative methods of constant comparison and thematic analysis will be presented along with the results related to diagnosis, medical decisions, finances, death, and family.

